# Small Fiber Polyneuropathy May Be a Nexus Between Autonomic Nervous System Dysregulation and Pain in Interstitial Cystitis/Bladder Pain Syndrome

**DOI:** 10.3389/fpain.2021.810809

**Published:** 2022-01-04

**Authors:** Dylan T. Wolff, Stephen J. Walker

**Affiliations:** ^1^Department of Urology, Wake Forest School of Medicine, Winston-Salem, NC, United States; ^2^Wake Forest School of Medicine, Wake Forest Institute for Regenerative Medicine, Winston-Salem, NC, United States

**Keywords:** interstitial cystitis, bladder pain syndrome, autonomic nervous system, sympathetic, parasympathetic, dysautonomia, small fiber polyneuropathy

## Abstract

Interstitial cystitis/bladder pain syndrome (IC/BPS) is a highly heterogeneous chronic and debilitating condition which effects millions of women and men in the United States. While primarily defined by urinary symptoms and pain perceived to be emanating from the bladder, IC/BPS patients frequently have co-occurring conditions and symptoms, many of which affect diverse body systems related to autonomic nervous system function. The impact on the autonomic system appears to stem from increased sympathetic innervation of the urinary tract, along with increased systemic sympathetic tone and decreased parasympathetic tone. Concurrent with these findings is evidence for destruction of peripheral sympathetic innervation to the sweat glands which may relate to small fiber polyneuropathy. It is unknown to what degree the wider alterations in autonomic function are also related to destruction/alterations in the small fibers carrying autonomic innervation. This potential nexus is an important point of investigation to better understand the unclarified pathophysiology of interstitial cystitis/bladder pain syndrome, the numerous co-occurring symptoms and syndromes, and for the identification of novel targeted therapeutic strategies.

## Introduction

**Figure 1:**
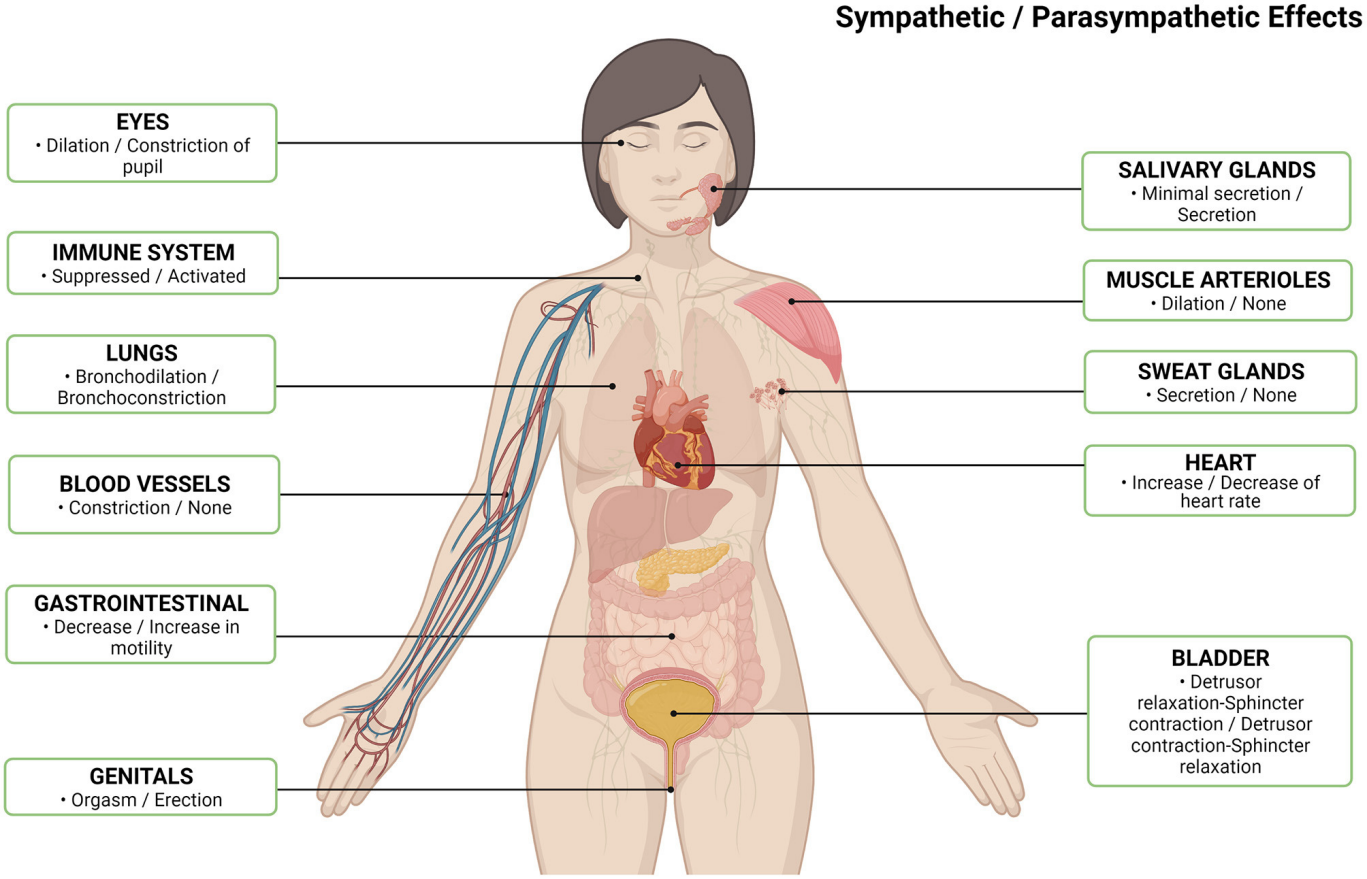
Normal autonomic nervous system effects per body system by sympathetic/parasympathetic (7). Created with Biorender.com.

Interstitial cystitis/bladder pain syndrome (IC/BPS) is a debilitating chronic affliction characterized by bladder pain of unspecified origin. Clinically, the American Urological Association defines IC/BPS as “an unpleasant sensation (pain, pressure, discomfort) perceived to be related to the urinary bladder, associated with lower urinary tract symptoms of more than 6 weeks duration, in the absence of infection or other identifiable causes” (1). It is estimated to affect 3.3–7.9 million women in the USA (2), up to an additional 2 million men (3), and costs over 750 million per year (4). Despite this societal and economic toll, IC/BPS continues to present as a highly heterogeneous condition with a poorly understood pathophysiology and variable treatment efficacy. Although bladder pain is the defining characteristic, IC/BPS frequently co-occurs with numerous non-urological symptoms and syndromes including psychiatric disorders such as depression, vague symptoms such as chronic fatigue and sleep disturbances, as well as other poorly understood chronic functional pain disorders such as irritable bowel syndrome (IBS), fibromyalgia syndrome (FMS), and migraines (5). The clustering of these comorbidities, along with evidence stemming from physiologic and anatomic studies, including the high frequency of small fiber polyneuropathy (SFPN) (6), point to widespread neurologic abnormalities in this patient population. Many of the symptomatic and functional changes described in patients with IC/BPS are related to the autonomic nervous system, i.e., the system responsible for the regulation of a wide variety of bodily systems and functions (Figure 1). Therefore, the objective of this review is to highlight and discuss the evidence for autonomic system dysregulation in IC/BPS patients and to explore the potential link between dysautonomia and SFPN.

### Urinary Bladder Changes in IC/BPS Patients Suggestive of Autonomic Nervous System Dysfunction

Focal bladder tissue in patients with IC/BPS appears to exhibit changes in autonomic innervation. Patient bladder biopsies, when compared to non-IC/BPS controls, show increased staining for tyrosine hydroxylase in the bladder wall, the enzyme catalyzing the principal step in catecholamine synthesis and a common marker for sympathetic nerves (8).

Beyond noradrenergic innervation, it has been shown that patients with IC/BPS have altered non-adrenergic non-cholinergic (NANC) autonomic staining in the bladder. These changes include an increase in the parasympathetic neurotransmitter vasoactive intestinal peptide (VIP) (9). Interpreted in the original study to be a marker for increased sympathetic innervation, more recent studies have found VIP to be present purely in parasympathetic neurons (8, 10). Another NANC, neuropeptide Y, has been found to be either elevated in IC/BPS bladders (9), or found in similar levels to those of control patients (11). This marker may be present in both sympathetic and parasympathetic fibers (8), with an unclear role in human bladder physiology (12). Finally, detrusor samples from IC/BPS patients display a decreased contractile response to acetylcholine, with an increased response to adenosine triphosphate (ATP) (13), which is co-transmitted by somatic and sympathetic neurons and affects numerous receptors (14). ATP is known to have numerous effects including mechanosensory, nociceptive, and inflammatory responses (15). Patients with IC/BPS are known to have increased purinergic sensitivity (10), with a receptor expression profile directed toward a pro-nociceptive and pro-inflammatory profile (15).

Associated with local tissue differences, evidence for altered autonomic signaling has also been found in the urine of IC/BPS patients. Compared to controls, IC/BPS patients have increased norepinephrine (with similar levels of normetanephrine) in their urine without any change from the effect of treatment, or their current symptomatic burden, indicating a consistency across IC/BPS patients (16). This finding of an elevation in urinary norepinephrine has been repeated in a smaller sample, and this group also found that patients with IC/BPS had higher levels of norepinephrine in their serum after resting while supine, but not while they were upright (17). Neither study tested for other catecholamines nor their metabolites.

Both serum and urinary norepinephrine are commonly used markers of sympathetic tone (18), with norepinephrine normally excreted by the kidneys both by glomerular filtration (19), as well as tubular secretion (20), with elevations indicating increased sympathetic tone. Norepinephrine is normally found in the serum stemming from sympathetic neurons innervating the vasculature (18), as well as secreted as part of the sympathetic baroreceptor response when upright which is cleared within 30 min of becoming supine. The elevated supine levels found in IC/BPS patients therefore indicate abnormally increased norepinephrine secretion at rest (21). The fact that there was no difference in normetanephrine levels is informative, as large amounts of systemic norepinephrine will be broken down into normetanephrine by extra-neuronal and systemic enzymes prior to secretion (18, 21). These findings, then, argue for a level of localized diffusion from the sympathetic nerves in the bladder wall.

Studies that use animal models have also reported increased local and systemic norepinephrine levels. In a feline model of IC/BPS, Feline Interstitial Cystitis (FIC), researchers found increased norepinephrine content in bladder wall samples as well as increased norepinephrine efflux from the bladder wall when electrically stimulated (22). Cats with FIC have also been shown to have elevated norepinephrine and dihydrophenylglycol (the primary intra-neuronal metabolite of norepinephrine) in their serum without concomitant increases in other catecholamines, their metabolites, adrenocorticotropic hormone, or cortisol (23), suggesting that these levels are predominantly from sympathetic innervation, and not from an increased adrenal response or total increased catecholamine synthesis.

It is unclear if these effects are causal in nature for IC/BPS, or a by-product of inflammation. Lipopolysaccharide (LPS) instillation into rat bladders causes increased sympathetic sprouting within the bladder wall (17), suggesting that the altered innervation may be caused by an inflammatory signal. Further, rats given large doses of norepinephrine to simulate increased sympathetic drive demonstrate increased urinary voiding, visceral sensitivity, and urothelial changes that mimic the thinning and inflammation of IC/BPS (17).

Taken together, these findings suggest that individuals with IC/BPS have altered neurologic innervation of their bladders. These patients appear to exhibit a combination of increased adrenergic sympathetic innervation with concomitant and non-specific NANC autonomic changes in bladder innervation. There is also increased urinary secretion of norepinephrine. Elevated serum levels argue for increased sympathetic tone throughout the body with concomitant filtration into the urine. However, the absence of systemic norepinephrine metabolites, as well as tissue studies showing increased sympathetic innervation, norepinephrine content, and secretion suggest that a portion of the levels found in the urine is due to local diffusion of norepinephrine from the bladder. Increased sympathetic innervation of the bladder in this context is initially puzzling as the normal bladder response to sympathetic innervation stems largely from β receptors, which induce relaxation and passive filling. This is contradictory to the typical symptoms experienced by patients with IC/BPS of painful bladder filling, urinary frequency, and nocturia. One possible explanation is that α receptors may induce contraction, although the extent to this function in humans is unknown (24).

### Systemic Changes in IC/BPS Patients Suggestive of Autonomic Nervous System Changes

Additional findings suggest autonomic changes that occur *outside of the urinary system* in patients with IC/BPS. Resting heart rate (HR) and blood pressure (BP) measurements are common methods to examine the autonomic tone of the body. The vagus nerve (parasympathetic) is responsible for decreasing the speed of the heart rate, while sympathetic tone increases the speed of the heart and raises blood pressure. Therefore, it is notable that those with IC/BPS have been found to have an elevated resting heart rate (25, 26) and diastolic blood pressure (26) at rest. Elevations in resting heart rate (without blood pressure changes) have also been seen prior to, while undergoing, and after undergoing a variety of mental stress models and was unrelated to perceived stress changes (27).

These changes in vascular autonomic function are further evidenced by the fact that while undergoing therapeutic hydrodistention, patients with IC/BPS are found to have significant elevations in blood pressure and heart rate that are not found in control patients (28, 29). This is likely an amplification of the known physiologic vesicovascular response wherein filling of the bladder induces a sympathetic reflex loop that increases heart rate and blood pressure (30). An exaggerated response can be inferred to indicate increased sympathetic outflow. Those undergoing spinal anesthesia did not experience these changes, providing further evidence that this is mediated through sympathetic loops in the spinal cord (29). It is also notable that at baseline prior to their operation, these patients *did not* have differences in HR or BP measurements compared to controls. These findings contrast with results from several groups that reported resting HR and BP are higher in those with IC/BPS (25–27).

Heart rate variability (HRV) testing, a standard measure of autonomic function, has been performed in several IC/BPS cohorts wherein the beat-to-beat variability of changes in heart rate can inform levels of autonomic tone to the cardiac system. These studies have predominantly found evidence for decreased cardiac parasympathetic tone (26, 31), and one suggested increased sympathetic tone without change to parasympathetic tone (17). It is worth noting that a recent meta-analysis by Koenig et al. on multiple chronic pain conditions, including IBS and FMS (frequently comorbid with IC/BPS), found similar alterations in HRV and suggest that vagal withdrawal is an important aspect of central pain sensitization (32).

Finally, those with IC/BPS have a decreased sudomotor response (peripheral sweat gland response to acetylcholine) (25), which is definitive evidence of destruction/malfunction of peripheral sympathetic function. This group also found that IC/BPS patients had higher rates of orthostatic intolerance (symptoms) compared to controls, however there were no differences in the rates of those found with positional orthostatic tachycardia syndrome (POTS), orthostatic hypotension, or tilt table mediated syncope revealing no evidence of cardiovascular autonomic damage. This is interesting as the same group found orthostatic symptoms and syncope to be highly self-reported by IC/BPS patients (5).

In sum, the systematic evidence for autonomic differences in IC/BPS patients indicates an increase in cardiovascular sympathetic tone, a decrease in cardiovascular parasympathetic tone, and additional evidence suggesting peripheral and superficial autonomic destruction in the absence of any proximal cardiovascular autonomic damage.

### Autonomic System Dysfunction and Small Fiber Polyneuropathy in IC/BPS

A significant proportion (49%) of women with FMS, one of the most common co-occurring conditions reported in patients with IC/BPS, have definitive skin biopsy findings consistent with SFPN (severely decreased superficial cutaneous nerve density) (33). The underlying pathophysiology of SFPN is complex, with a multitude of known causes including familial/genetic syndromes, metabolic disorders including diabetes mellitus, autoimmunity (Sjogren's syndrome being the most common), and more (34). However, up to 50% of cases are idiopathic, without a known cause (35). The fibers implicated in SFPN, αδ fibers as well as unmyelinated c fibers, provide sensory innervation not only to the skin, but also provide autonomic innervation to the viscera. It is unsurprising that those with idiopathic SFPN have decreased autonomic nerve fiber density to the skin (36) and high levels of autonomic symptoms (37).

The high co-occurrence of SFPN with FMS has raised the question of the role of small fiber loss in the etiology of the diverse array of symptoms found in FMS (34, 38). SFPN may present in a patchy and inconsistent manner including microscopic microvasculopathy that has been proposed to cause impaired muscle perfusion with consequent deep muscle aches and fatigue, as well as impaired gastrointestinal perfusion leading to abdominal pain and other gastrointestinal symptoms (34). In fact, SFPN+ women with FMS have more microvascular autonomic symptoms than those without (39).

Several small case series studies have reported a similarly high incidence, up to 63%, of SFPN in women with IC/BPS (6, 40), even when controlling for comorbid FMS. Further, several of the other disorders, beyond FMS, that commonly co-occur with IC/BPS have also been found to exhibit high rates of SFPN. These include some forms of POTS (41), complex chronic pelvic pain (CPP) (42), and chronic fatigue syndrome (CFS) (43) indicating a pathophysiologic link between these syndromes. The fact that many patients do not have evidence for SFPN argues that it is not the originating cause of these diseases. It may play a role initiating the disease only in a subset of patients or arise later in their disease process. In none of these cases is the underlying cause of SFPN known.

Currently, the role that co-occurring SFPN may play in the diverse and systemic symptoms that patients with IC/BPS experience is actively being investigated. It is known that patients with IC/BPS show evidence of peripheral sympathetic neuropathy (25). As SFPN may be patchy and non-length dependent in presentation, it is possible that reported skin findings underrepresent the true prevalence of SFPN. What is not known is whether the underlying prevalence of SFPN plays an explanatory or contributory role to the many other common comorbidities found.

At this time, much of autonomic testing is validated on a diabetes model, wherein diabetes causes a length-dependent and predictable progression of neuropathy. Cardiac testing in these same patients can also reliably predict other forms of more peripheral autonopathy (44). SFPN, on the other hand, may be inconsistent in distribution and non-length dependent (35). A skin biopsy-based finding of SFPN+ is not correlated with autonomic changes reflected in a sensitive and specific test for autonomic failure—the composite autonomic severity score (CASS) (45)- used in other models.

**Figure 2:**
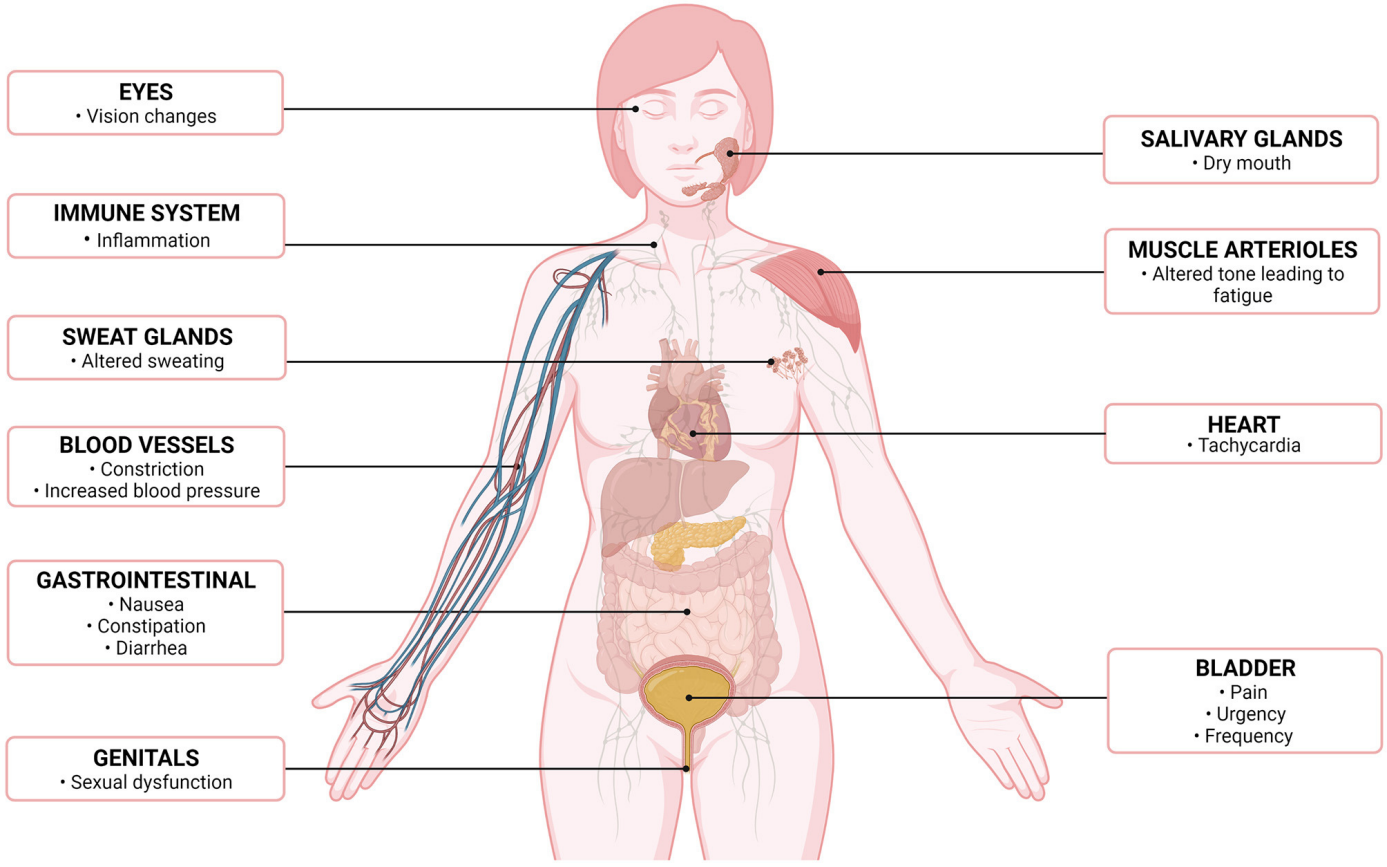
Altered autonomic symptoms/effects, by organ system, in patients with interstitial cystitis/bladder pain syndrome (7). Created with Biorender.com.

Common symptoms found in patients with IC/BPS such as vision changes (46), dry mouth (47), sexual dysfunction (48), dyspepsia, nausea (5, 46, 47, 49), and constipation (50) are consistent with dysautonomia but have not been specifically examined (Figure 2). None of the tests performed have evaluated the function of the autonomic system beyond the cardiovascular and sudomotor systems. Therefore, it is unlikely that the testing discussed in this review would have offered explanations for the autonomic dysfunction these patients are experiencing. Further, SFPN is not universally found in IC/BPS patients, nor are autonomic symptoms. Whether studies have failed to find structural autonomic defects in most cases due to underpowering of studies (too low number of patients with SFPN) is unknown. Moreover, their patchy and inconsistent presentation could additionally underpower studies as the individual autonomic systems that are dysregulated or damaged likely differ from patient to patient. Therefore, the autonomic symptoms may be a consequence of SFPN, but it was not able to be adequately studied/captured by the authors as at the time of their experiments there was no evidence compelling them to consider this angle.

## Discussion

The findings regarding autonomic system dysfunction in patients with IC/BPS may initially appear to be unrelated. Abnormal findings include an increased sympathetic bladder innervation (11), non-specific altered NANC bladder innervation (9, 11, 13), increased urinary secretion of norepinephrine (16, 17, 51), as well as increased serum norepinephrine (17, 51). Furthermore, there are non-specific signs of increased systemic sympathetic tone through an increased resting heart rate (25–27) and blood pressure (26), and an increased vesicovascular response to bladder distension (28, 29). HRV testing, however, suggests decreased cardiac parasympathetic tone (26, 31). Finally, there is evidence of peripheral sympathetic autonopathy, in the absence of evidence for autonomic cardiovascular deficits (25). One reason for the lack of a clearer picture that defines the role of dysautonomia in IC/BPS is that no studies have comprehensively tested many of the symptoms which may be autonomic in origin, nor offer a compelling explanation for them.

Therefore, the reported findings regarding SFPN and autonomic function reflect two overlapping elements that are co-existing in patients with IC/BPS, one of altered/increased tone and function (histological differences, urinary secretion of catecholamines, altered vascular tone and dynamics, altered HRV), and another of overt destruction of peripheral autonomic fibers without widespread autonomic failure (e.g., abnormal sudomotor testing). SFPN, even with a high prevalence in IC/BPS, is unlikely to be the direct cause of the altered sympathetic/parasympathetic tone, at least insofar as we currently understand the pathology.

It is possible that SFPN and altered autonomic tone are largely unrelated. SFPN may be a co-occurring finding that is common in patients with IC/BPS, but which does not constitute a direct cause or consequence of the disease, while the altered autonomic tone may be related to the chronic pain/stress of the disease, or as a factor initiating some of the bladder dysfunction. However, there is reason to believe that the two may be related.

SFPN is commonly associated with autoimmune disorders (34). IC/BPS is also commonly theorized to be autoimmune in nature, stemming from numerous comorbid autoimmune diseases, a high frequency of autoantigens, and the use of induced autoimmune cystitis in animals as a model for IC/BPS (52, 53). The association of SFPN with IC/BPS invigorates this debate and should be further explored. Further investigation of this link is critical not only for the better understanding of the underlying pathophysiology, but also for potential treatment models. IC/BPS has no universally effective therapies, and the process of finding adequate symptom management is often one of trial and error (1). Cases of SFPN related to autoimmune disease have been successfully treated with IVIG (54). If SFPN is causally related to the pain/symptoms of IC/BPS, then this implicates a potential treatment target which has not yet been exploited and has the potential to be a critical tool in helping relieve the symptomatic burden of these patients.

The available evidence cannot distinguish if sympathetic upregulation, parasympathetic downregulation, or both are an initiating factor in the disease process, or if they are consequences of it. One possible link is that the autonomic nervous system is intricately tied to inflammation, with the parasympathetic system being dubbed the “cholinergic anti-inflammatory pathway” (55). Downregulation of the parasympathetic nervous system is commonly found within autoimmune diseases and is even found to precede the onset of rheumatoid arthritis (RA). Further evidence is given by the fact that vagal stimulation has shown to have anti-inflammatory effects in this population (56). This insight offers to link our seemingly disparate findings of altered autonomic *tone* with limited destruction of autonomic *function* and the chronic inflammation and pain present in IC/BPS. Even if IC/BPS is not autoimmune in nature, this highlights a connection between the pain and inflammation present and the autonomic changes noted.

Similarly, it has been proposed that increased chronic stress and sympathetic tone in IC/BPS may play a causative role in its pathogenesis (51). In this scenario, the bladder is hypersensitized from increased physiologic stress/sympathetic tone which causes mastocytosis, a leaky bladder epithelium, and a vicious cycle wherein subsequent pain and voiding dysfunction further perpetuates stress and sympathetic tone. Is it possible that this sort of autonomic dysregulation similarly changes the superficial innervation of the skin leading to the findings of SFPN? While it is unclear what the initiating factor is, it is probable that the findings of autonomic dysfunction are linked to the pathophysiology of IC/BPS.

In conclusion, there is strong evidence of an increased sympathetic tone and decreased parasympathetic tone among patients with IC/BPS, and some additional evidence for overt sympathetic destruction. However, while many symptoms that could be attributed to dysautonomia are reported by IC/BPS patients, few have been conclusively established in the laboratory setting. A significant proportion of patients with IC/BPS also have a definitive finding of SFPN in their distal limbs, and some patients have established distal sweating irregularities consistent with autonomic damage to the skin. Because these symptoms, related both to autonomic system function and neuropathy, are heterogeneous, and their respective underlying etiologies are likely multi-factorial, drawing definitive conclusions regarding cause-and-effect relationships are not possible at this time. Investigators are encouraged to continue to explore the overlap between SFPN and the autonomic system in patients with IC/BPS so that we may better understand the pathophysiology and identify meaningful therapeutic targets and strategies.

## Author Contributions

DW initiated concept of the article, performed literature review, and wrote the manuscript. SW provided expertise and guidance for the scientific and writing process, cowrote the manuscript, and edited the manuscript. All authors contributed to the article and approved the submitted version.

## Funding

This work was supported by R01 DK124599: Molecular Characterization of a Large Cross-Sectional and Longitudinal Collection of Patients to Investigate Disease Progression in IC/BPS.

## Conflict of Interest

The authors declare that the research was conducted in the absence of any commercial or financial relationships that could be construed as a potential conflict of interest.

## Publisher's Note

All claims expressed in this article are solely those of the authors and do not necessarily represent those of their affiliated organizations, or those of the publisher, the editors and the reviewers. Any product that may be evaluated in this article, or claim that may be made by its manufacturer, is not guaranteed or endorsed by the publisher.
